# No effect of epoprostenol on right ventricular diameter in patients with acute pulmonary embolism: a randomized controlled trial

**DOI:** 10.1186/1471-2466-10-18

**Published:** 2010-03-30

**Authors:** Albertus J Kooter, Richard G IJzerman, Otto Kamp, Anco B Boonstra, Yvo M Smulders

**Affiliations:** 1 Department of Internal Medicine, VU University Medical Center, Amsterdam, the Netherlands; 2 Department of Cardiology, VU University Medical Center, Amsterdam, the Netherlands; 3 Department of Pulmonology, VU University Medical Center, Amsterdam, the Netherlands

## Abstract

**Background:**

Right ventricular dilatation in the setting of acute pulmonary embolism is associated with an adverse prognosis. Treatment with a pulmonary vasodilator has never been studied systematically. We evaluated the effect of epoprostenol on right ventricular diameter and function in patients with acute pulmonary embolism and right ventricular dilatation.

**Methods:**

In a randomized, single-blind study, 14 patients with acute pulmonary embolism received epoprostenol or placebo infusion for 24 hours on top of conventional treatment. Effects on right ventricular end-diastolic diameter, systolic pulmonary artery pressure, right ventricle fractional area changeand tricuspid annular plane systolic excursion were assessed by serial echocardiography. Furthermore Troponin T and NT-proBNP were measured serially.

**Results:**

Compared to placebo, epoprostenol was associated with a relative change from baseline in right ventricular end-diastolic diameter of +2% after 2.5 hours and -8% after 24 hours. Epoprostenol did not have a significant effect on systolic pulmonary artery pressure, right ventricular fractional area change and tricuspid annular plane systolic excursion, nor on biochemical parameters.

**Conclusion:**

In patients with acute pulmonary embolism and right ventricular overload, treatment with epoprostenol did not improve right ventricular dilatation or any other measured variables of right ventricular overload.

**Trial Registration:**

*Registration*: URL: NCT01014156

*Medical ethical committee*: Medisch-ethische toetsingscommissie (METc) from the VUmc (free university medical centre)

## Background

Pulmonary embolism is a frequent and potentially fatal disorder. Despite immediate treatment with anticoagulants, morbidity and mortality are still high when hemodynamically stable patients with pulmonary embolism have echocardiographic signs of acute right ventricular overload [1-3].

An acute increase in right ventricular afterload is the hallmark of severe pulmonary embolism, and is responsible for many of its clinical manifestations and complications. The traditional view is that mechanical obstruction by thrombus mass causes pulmonary hypertension. Therefore, treatment focusses on relieving mechanical obstruction, either by anticoagulation if patients are stable, or by thrombolytic therapy in case of hemodynamic instability [4]. The optimal treatment for haemodynamically stable patients with signs of right ventricular overload is unclear. Despite one clinical trial showing some benefit of thrombolysis in normotensive patients with pulmonary embolism and echocardiographic signs of right ventricular dysfunction [5], thrombolytic therapy continues to be highly controversial in this patient category [6-8].

Apart from mechanical obstruction, vasoconstriction of the pulmonary vasculature plays a pivotal role in the acute rise in pulmonary artery pressure in patients with pulmonary embolism [9]. This is supported by the marked discrepancy between hemodynamic manifestations of acute pulmonary embolism and the degree of mechanical obstruction [10]. Furthermore, bringing about a strictly mechanical obstruction in a pulmonary artery causes only a modest rise in pulmonary artery pressure (PAP), rarely resulting in right-sided heart failure, whereas pulmonary embolism with obstruction of only 25% of the pulmonary vascular tree can cause marked acute pulmonary hypertension [11]. Finally, studies in experimental animal models support a crucial role for pulmonary vasoconstriction in acute pulmonary embolism [12,13].

Given the role of pulmonary vasoconstriction in the pathogenesis of pulmonary embolism-associated pulmonary hypertension, the potential benefit of pulmonary vasodilators is important. There is experimental evidence suggesting that antagonising pulmonary vasoconstriction by administration of selective vasodilators is beneficial. In animal models of acute pulmonary embolism prostacyclin, a relatively selective pulmonary vasodilator, prevented or partially reversed the rise in pulmonary vascular resistance and pressure [14,15]. Pretreatment with a prostacyclin analogue protected mice from pulmonary embolism-related death in a dose-dependent manner [16]. In humans, a case report suggested that inhaled prostacyclin may also be beneficial for acute pulmonary hypertension associated with pulmonary embolism [17]. However, the potentially beneficial role of pulmonary vasodilatory therapy in acute pulmonary embolism has never been studied in a systematic way.

In this randomized, controlled trial, we evaluated the effects of epoprostenol, the pharmacological form of prostacyclin, on echocardiographic abnormalities, cardiac biomarkers and on hemodynamic and respiratory symptoms in patients with pulmonary embolism with echocardiographic signs of right ventricular overload.

## Methods

### Subjects

A total 82 patients with acute pulmonary embolism, as diagnosed by spiral CT, were screened for eligibility. Patients were potentially eligible if they had a recent (<24 hours) onset or progression of symptoms, and if the clinical probability of finding right ventricular overload by echocardiography was high, as indicated by one of the following symptoms or signs: (near) collapse, systolic blood pressure <100 mmHg, diastolic blood pressure <60 mmHg, pulse rate >100 beats/min, elevated jugular venous pressure, electrocardiographic abnormalities (pulmonary P-waves, S1Q3-complex, right bundle branch block, T-wave inversions in precordial leads), tachypnea (breathing frequency >20/min), PaO_2 _<80 mmHg (with oxygen supplementation of 2 liters), peripheral oxygen saturation (SaO_2_) <95% or dyspnea [2,3].

After informed consent was obtained, patients were screened by transthoracic echocardiography for signs of pulmonary hypertension. If acute right ventricle dilatation was present (right ventricular end-diastolic diameter (RVED) >30 mm [2,18] in the absence of substantial right ventricular hypertrophy (free wall thickness <7 mm)) or if systolic pulmonary hypertension (systolic PAP >40 mmHg) was present, patients were included in the study. During echocardiography all patients received 2 liters of oxygen supplementation. Patients were excluded if they refused or were unable to give informed consent, or if one of the following was present: age <18 years, pregnancy, body mass index >35 kg/m^2^, circulatory shock (blood pressure <80/45 mmHg), mechanical ventilation, treatment with thrombolytic therapy, placement of a vena cava filter, atrial fibrillation or severe pre-existent cardiopulmonary disease (heart failure, obstructive pulmonary disease, emphysema).

The study protocol was approved by the local ethics committee (METc), and written informed consent was obtained from each patient prior to study entry.

### Study design

Patients were randomized to receive intravenous epoprostenol sodium (Flolan^®^; GSK; Zeist, the Netherlands) or saline placebo infusion. Infusion of epoprostenol was started in a dose of 1 ng/kg/min. Every 10 minutes the dose was increased with 1 ng/kg/min to a maximum dose of 4 ng/kg/min. Patients not randomised to epoprostenol received a similar syringe pump containing isotonic saline. During titration of epoprostenol infusion, blood pressure, pulse rate and peripheral oxygen saturation were measured at 5 minute intervals. If, during titration, mean blood pressure dropped by >10 mmHg, the dose of epoprostenol was not further increased and 0.5 liter of extra sodium chloride was administrated in 30 minutes. If blood pressure dropped by >15 mmHg or if oxygen saturation dropped by >7% (or absolute <85%) epoprostenol infusion was decreased to the previous titrated dose. If blood pressure and/or oxygen saturation were not restored by down-titration, epoprostenol infusion was stopped. After inclusion of the first 8 subjects, no side effects of epoprostenol treatment at a dose of 4 ng/kg/min were observed. Because our study is a proof-of-principle study we decided to increase epoprostenol infusion rate every 10 minutes with 1 ng/kg/min to the maximum dose of 8 ng/kg/min, which is substantially higher than maximum initial doses used in epoprostenol intervention trials in patients with chronic pulmonary hypertension [19]. After reaching the maximum dose, blood pressure, pulse rate and SaO_2 _were assessed hourly for the entire duration of epoprostenol or placebo infusion. Epoprostenol infusion was continued for 20 hours, then tapered gradually during 4 hours and stopped. Hence, total treatment with epoprostenol or placebo encompassed a period of 24 hours.

The patient, the technician performing the echocardiographic examinations, as well as the cardiologist assessing the echocardiographic recordings were blinded for treatment allocation. The physician providing clinical care and monitoring vital parameters was not blinded in order to be able to respond appropriately if the patient showed signs of hemodynamic or respiratory deterioration.

All patients were treated with low-molecular-weight heparin adjusted for body weight, acenocoumarol, supplemental oxygen (2 l/min) and a isotonic saline infusion (80 ml/hr).

### Study Evaluations

At baseline, after 2.5, 24 and 72 hours echocardiographic parameters were obtained. At baseline, after 4, 24 and 72 hours serum creatine kinase, cardiac troponin T and NT-proBNP were measured. At baseline, after 4 and 24 hours, arterial blood gas analysis was performed to detect hypoxemia or a rise in PaCO_2 _as a sign of intrapulmonary shunting due to epoprostenol infusion. The primary end point was the change in RVED between baseline measurement and during treatment (i.e. measurements 2 and 3). Secondary end points were right ventricular diameter 48 hours after treatment was stopped (i.e. measurement 4), systolic PAP, tricuspid annular plane systolic excursion (TAPSE), right ventricular fractional area change (RVFAC), serum cardiac troponin T and NT-proBNP.

### Echocardiographic Studies

Standard two-dimensional and Doppler echocardiography was performed with a commercially available Vivid 7 system (GE medical systems; Horten, Norway) using a 3.0 Mhz broadband (M3S) transducer; serial echocardiograms were analysed off-line. All examinations included standard parasternal, apical and subcostal views and were stored digitally for subsequent analysis. Right ventricular function was assessed by TAPSE and RVFAC, right ventricular dimensions by long axis and tricuspid annular size from the apical 4 chamber view. Furthermore, estimation of systolic PAP was obtained by peak tricuspid regurgitant jet with continuous wave Doppler and adding the right atrial pressure derived from the collapse index of the inferior vena cava [20]. Cardiac output (left ventricular outflow traject) was measured with pulse-doppler. The two-dimensional echocardiograms were interpreted by two experienced echocardiographers.

### Laboratory Studies

NT-proBNP concentrations were determined with an immunoradiometric assay kit (Roche diagnostics, module E170; Mannheim, Germany). Normal values are depending on age and gender, but in general a NT-proBNP <200 pg/ml is considered normal. Cardiac troponin T concentrations were determined using a microparticle enzyme immunoassay (Roche Diagnostics, Module E170; Mannheim, Germany). A normal value was considered a troponin T <0.04 ng/ml.

### Statistical Analyses

All analyses were performed using the SPSS statistical software package (version 15; SPSS; Chicago, IL). Data are shown as mean ± SD or median (range). Biochemical parameters were log-transformed before analysis. The Student-t test was used to compare RV parameters between treatment arms and to compare relative change of RVED compared to baseline between treatment arms. Students-t test was chosen primarily to calculate confidence intervals which may be appropriate for power analysis for future studies. Moreover, due to small sample sizes and non-normal distributions of biochemical parameters, also a nonparametric test (Mann-Whitney *U*) was performed.

Differences were considered significant at p < 0.05. This study was intended as a safety and proof-of-concept study. In addition, obtaining point estimates of effect-size of epoprostenol on primary endpoints may be of use for the design of future studies. Hence, the study is designed as a pilot study, and does not include a formal power analysis based on clinically relevant effect sizes.

## Results

### Subjects

**Figure 1:**
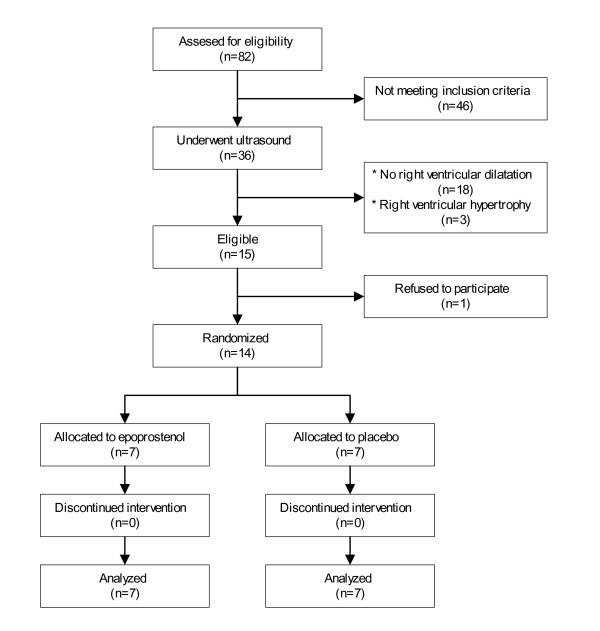
**Patient flow**.

Eighty two patients were screened and 14 patients were randomized to either epoprostenol (n = 7) or placebo (n = 7; Figure 1). The baseline characteristics of the treatment groups were similar, as shown in Table 1. In both groups treatment was started on average 14 hours after the onset or progression of symptoms. All active treatment patients received at least 4 ng/kg/min of epoprostenol. The 3 patients who were treated with increasing dose received 8.0, 8.0 and 5.6 ng/kg/min epoprostenol respectively. The dose in the last patient could not be raised due to facial flushing and headache. In two patients in the placebo group, the measurements at 72 hours, including echocardiography, could not be performed because of logistical reasons.

**Table 1:** Characteristics of Subjects at enrollment*

Characteristics	Placebo (n = 7)	Epoprostenol (n = 7)
Male gender	4 (57)	4 (57)
Age, yr	54.4 (19.9)	55.3 (18.2)
Weight, kg	78.9 (11.2)	74.0 (9.7)
RVED, cm	4.1 (0.84)	4.1 (0.83)
Systolic PAP, mmHg	47 (10.6)	57 (13.9)
Right ventricle free wall diameter, mm	6.1 (0.7)	5.9 (0.9)
NT-proBNP, pg/ml	1467 (39-8549)	3885 (270-12339)
Troponin T, ng/ml	0.01 (0.01-0.13)	0.03 (0.01-0.12)
PaO_2_, mmHg	85 (14)	74 (12)

*Definition of abbreviations*: PAP = pulmonary artery pressure; RVED = right ventricular end-diastolic diameter; PaO_2 _= partial arterial oxygen pressure

*Data are presented as No. (%) or mean (SD). NT-proBNP and troponin T are presented as median (range)

### Effects on Right Ventricle End-Diastolic Diameter

**Figure 2:**
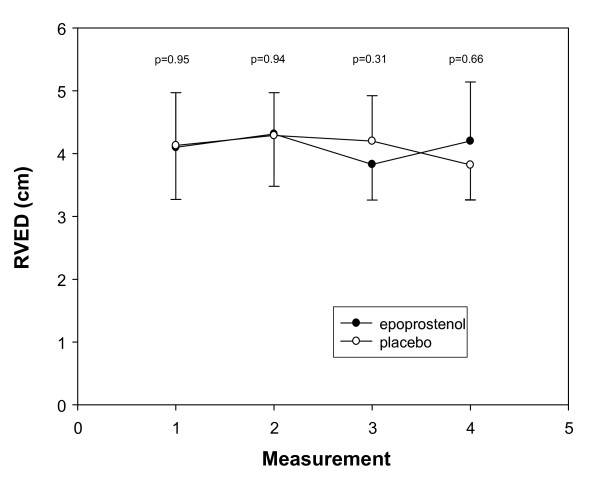
**Mean (± SD) right ventricular end-diastolic diameter (RVED) for both treatment groups at baseline, 2.5 and 24 hours after initiation of treatment and 48 hours after treatment was stopped (measurement 1, 2, 3 and 4, respectively)**.

At baseline, the mean RVED was similar in both treatment groups (4.10 ± 0.83 cm in the epoprostenol arm and 4.13 ± 0.84 cm in the placebo arm). Epoprostenol did not have a significant effect on RVED after 2.5 and 24 hours (Figure 2). After 2.5 hours, the relative change in RVED compared to baseline was similar in both treatment groups, +4.2% in the placebo arm and +6.1% in the epoprostenol arm (difference in change compared to baseline +1.9% (95% confidence interval, -5.8 to 9.6, *P *= 0.60). After 24 hours, RVED had increased by 2.4% in the placebo arm and had decreased by 5.6% in the epoprostenol arm (difference in change compared to baseline -8.0% (95% confidence interval, -21.2 to 5.2, *P *= 0.21)). Results were similar for the 3 patients in whom epoprostenol was titrated to a mean dose of 7.2 ng/kg/min (data not shown).

### Other Efficacy End Points

**Figure 3:**
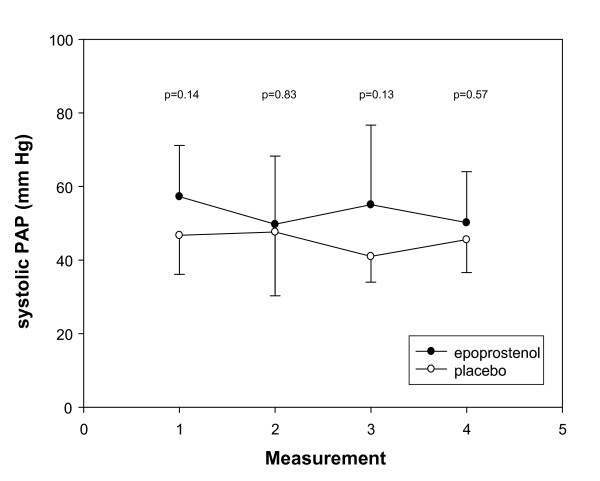
**Mean (± SD) systolic PAP for both treatment groups at baseline, 2.5 and 24 hours after initiation of treatment and 48 hours after treatment was stopped (measurement 1, 2, 3 and 4 respectively)**.

Measurements of systolic PAP, TAPSE, RVFAC, end-diastolic right atrial diameter, end-diastolic right/left ventricular ratio and biochemical parameters of right ventricular overload are presented in Table 2. All echocardiographic parameters of right ventricular function remained stable during follow up. Biochemical paramaters improved in both groups. At no time point, significant differences of echocardiographic and biochemical parameters between treatment groups were observed (*P *> 0.20). Systolic PAP at baseline and follow up for both treatment groups is presented in Figure 3.

**Table 2:** Changes in echocardiographic parameters, blood pressure and biochemical variables associated with acute right ventricular overload

		Baseline	T = 2.5 hrs	T = 24 hrs	T = 72 hrs
**RVED (mm)**	**Placebo**	41 ± 8	43 ± 8	42 ± 7	38 ± 5
	**Epoprostenol**	41 ± 8	43 ± 7	38 ± 6	40 ± 9
**Systolic PAP (mmHg)**	**Placebo**	47 ± 11	48 ± 17	41 ± 7	46 ± 9
	**Epoprostenol**	57 ± 14	50 ± 19	55 ± 22	50 ± 14
**TAPSE (cm)**	**Placebo**	2.0 ± 0.5	2.1 ± 0.6	2.3 ± 0.3	2.2 ± 0.6
	**Epoprostenol**	1.9 ± 0.6	1.9 ± 0.5	2.0 ± 0.6	2.0 ± 0.5
**RVFAC (%)**	**Placebo**	24 ± 18	28 ± 21	25 ± 17	29 ± 16
	**Epoprostenol**	34 ± 24	34 ± 10	35 ± 16	33 ± 10
**RAD (cm)**	**Placebo**	4.7 ± 0.7	4.4 ± 0.8	4.7 ± 1.3	4.3 ± 0.9
	**Epoprostenol**	4.2 ± 0.9	4.6 ± 1.0	4.1 ± 1.2	4.1 ± 1.0
**RV/LV**	**Placebo**	0.82 ± 0.16	0.88 ± 0.10	0.89 ± 0.15	0.69 ± 0.07
	**Epoprostenol**	0.87 ± 0.24	0.97 ± 0.14	0.77 ± 0.23	0.79 ± 0.15
**ABPs (mmHg)**	**Placebo**	117 ± 6	114 ± 12	118 ± 19	124 ± 11
	**Epoprostenol**	125 ± 27	123 ± 26	118 ± 18	134 ± 29
**ABPd (mmHg)**	**Placebo**	70 ± 10	71 ± 6	72 ± 12	74 ± 11
	**Epoprostenol**	66 ± 14	70 ± 12	71 ± 11	72 ± 11
**ABPm (mmHg)**	**Placebo**	86 ± 10	86 ± 7	87 ± 11	91 ± 10
	**Epoprostenol**	82 ± 15	88 ± 6	87 ± 12	93 ± 12
**CO (l/min)**	**Placebo**	6.2 ± 1.7	6.5 ± 0.6	6.7 ± 1.2	5.4 ± 1.2
	**Epoprostenol**	5.5 ± 1.2	6.4 ± 1.1	5.9 ± 0.8	6.0 ± 0.8
**NT-proBNP (pg/ml)**	**Placebo**	1467 (39-8549)	925 (33-8322)	694 (84-6954)	343 (130-7240)
	**Epoprostenol**	3885 (270-12339)	3043 (352-4811)	3089 (415-4892)	614 (147-4268)
**Troponin T (ng/ml)**	**Placebo**	0.01 (0.01-0.09)	0.01 (0.01-0.09)	0.01 (0.01-0.07)	0.01 (0.01-0.04)
	**Epoprostenol**	0.03 (0.01-0.13)	0.02 (0.01-0.12)	0.01 (0.01-0.07)	0.01 (0.01-0.08)

*Definition of abbreviations*: PAP = pulmonary artery pressure; TAPSE = tricuspid annular plane systolic excursion; RVFAC = right ventricular fractional area change; RAD = right atrial end-diastolic diameter; RV/LV = right/left ventricular end-diastolic diameter; ABPs = systolic arterial blood pressure; ABPd = diastolic arterial blood pressure; ABPm = mean arterial blood pressure; RVED = right ventricular end-diastolic diameter;

Values are given as the mean ± SD. NT-proBNP and troponin T are given as median (range).

At no time point significant differences of these parameters between treatment groups were observed (*P *> 0.20). Data for NT-proBNP and Troponin T were log-transformed for statistical testing.

### Adverse events

There were no serious adverse events in either treatment arm. Two patients showed facial flushing during infusion with epoprostenol, however, this was transient and did not influence infusion rate. As described above, in one patient administraton of epoprostenol could not be increased to a maximum 8 ng/kg/min because of persistent facial flushing and headache. Symptoms disappeared after gradual lowering of the infusion rate. Parameters of pulmonary gas exchange (PaO_2_, PaCO_2 _or SaO_2_) were similar at baseline and did not significantly change during treatment (data not shown). There were no blood pressure changes necessitating fluid resuscitation or lowering of the epoprostenol infusion rate.

## Discussion

This is the first study to investigate the effect of a vasodilator for treatment of acute pulmonary embolism in a randomized controlled trial. Our study suggests that adding epoprostenol for 24 hours to standard treatment of acute pulmonary embolism in patients with right ventricular dilatation does not result in a significant change in right ventricular overload.

Clearly, the sample size is too small to detect relatively small effects of epoprostenol. However, it should be emphasized that the point estimate of any treatment effect of epoprostenol on right ventricular diameter and function is very small: after 2.5 hours 2% in favour of placebo, after 24 hours 8% in favour of epoprostenol. These small changes are not likely to be clinically relevant. Estimation of relevant degrees of RVED or PAP reduction can possibly be derived indirectly from human thrombolysis studies, since thrombolytic treatment was associated with reduced mortality (4.7% vs 11.1%) in the setting of right ventricular dilatation in acute pulmonary embolism [21]. Three studies reported data on RVED after thrombolytic treatment. These studies demonstrated reductions in right ventricular dimensions ranging from 15% to 49% within 24 hours [22-24]. Combining these results, a decrease in RVED of at least 15% might be the best possible estimation of clinical relevant reduction in RVED. This is twice the maximal observed effect we found in our pilot study. A post-hoc power analysis of our study data indicated that we had 80% power to detect a difference in RVED between treatment arms as large as 16%. Furthermore, it would require 55 subjects to confirm an 8% difference in treatment effect with a study of sufficient statistical power (80%). In considering such a trial, however, one would need to weigh the objective of demonstrating only a potential modest benefit in mortality reduction against the potential safety concerns and the costs of epoprostenol treatment.

Why doesn't epoprostenol have a more pronounced effect? In animal models of acute pulmonary embolism, treatment with different vasodilators, including prostacyclin, was associated with a more pronounced decrease in mean PAP of 12-33% [14,25-27]. However, the administration of vasodilating agents in these experiments was started immediately after or even before the onset of acute pulmonary embolism. Since pulmonary vasocontriction is an early and partially transient phenomenon in acute pulmonary embolism [14], this might explain why animal models show a larger benefit of vasodilator treatment. In our pilot study, we treated patients with epoprostenol several hours after the onset of symptoms. The absence of a decrease in systolic PAP in either treatment group may indicate that patients were already past the phase of acute pulmonary vasocontriction. Normally, it takes time to get to the hospital, to undergo diagnosis and echocardiography, making earlier treatment difficult to realise in normal practice. This may also be the explanation for the minimal effect of the serotonin receptor blocker ketanserin on PAP and cardiac output in patients with acute pulmonary embolism [28]. For inpatients suffering from acute pulmonary embolism, however, the situation might be different, and pulmonary vasodilatory therapy may still be effective. It might be argued that right ventricular dysfunction was not severe enough to expect a beneficial effect of epoprostenol. Indeed, TAPSE was at the lower limit of normality, suggesting at most mild right ventricular dysfunction. Pseudonormality of TAPSE can be caused by severe tricuspid valve regurgitation but this was not the case in our patients. RVFAC, however, another indicator of right ventricular function, was diminished suggesting at least moderate dysfunction. It is intriguing why TAPSE and RVFAC seem discongruent in our patients. These parameters usually correlate well, although TAPSE can be normal in patients with a low RVFAC [29]. It is, however, possible that a pulmonary vasodilator would have a more pronounced effect in patients with more severe right ventricular dysfunction.

The choice for intravenous epoprostenol as a vasodilator merits discussion. Firstly, it could be argued that intravenous administration of epoprostenol might cause nonselective vasodilation which could lead to intrapulmonary shunting and systemic hypotension. However, hypotension or a decrease in PO_2 _(data not shown) did not occur in the patients who received epoprostenol infusion in our study. Inhalation of prostacyclin or nitric oxide is more selective for pulmonary vasculature and has shown to decrease pulmonary artery pressure in case reports of acute pulmonary embolism [30,31]. However, inhalation of prostacyclin might increase dead space, possibly due to mucosal swelling [32] and inhalation of nitric oxide is difficult to implement in patients who are not on mechanical ventilation. Secondly, it has been suggested that intravenous administration of epoprostenol in pigs was associated with reduced right ventricular contractility [33], yet a recent study could not detect a change in measures of right ventricular contractility [34]. In humans with chronic pulmonary hypertension, epoprostenol was associated with unchanged right ventricular dimensions or even increased cardiac output [35,36]. The effect of epoprostenol on right ventricular function in acute pulmonary hypertension is not well known. Thirdly, it is possible that the dose of epoprostenol was not sufficient to decrease PAP. One might argue that uptitrating iv epoprostenol until systemic side effects like arterial hypotension occur, would be more likely to reveal beneficial effects on right ventricular overload and pulmonary pressures. However, increasing the dose of epoprostenol to 7.2 ng/kg/min, which is generally considered as high-dose, in 3 patients did not result in reduction in RVED. We did not further increase epoprostenol infusion rate because higher doses are usually associated with intolerable side-effects [37]. Finally, it is also important to point out that other pulmonary vasodilators have different pharmacological properties, and our findings thus cannot be extrapolated to these alternatives.

## Conclusions

This small randomized controlled trial investigated the effect of epoprostenol in patients with acute pulmonary embolism and right ventricular dilatation. In contrast to the results of several case reports and studies in animals, treatment with epoprostenol in humans did not improve right ventricular dilatation or any other measured variables of right ventricular overload.

## Competing interests

Albertus J Kooter has no conflicts of interest to disclose; Richard G IJzerman has no conflicts of interest to disclose; Otto Kamp has no conflicts of interest to disclose; Anco B Boonstra's employer received in the past 3 years an unrestricted educational grant from GSK and Actelion, and has been reimbursed for doing phase III trials for GSK and Actelion. Anco Boonstra has served as a paid speaker for Actelion; Yvo M Smulders has no conflicts of interest to disclose.

## Authors' contributions

AJK was involved with data acquisition, participated in the design of the study, performed the statistical analysis and drafted the manuscript; RGIJ participated in the statistical analysis and manuscript drafting; OK carried out the echocardiographic analysis; ABB participated in the design of the study; YSM supervised the design of the study, participated in the statistical analysis and manuscript drafting. All authors read and approved the final version of the manuscript.

## Pre-publication history

The pre-publication history for this paper can be accessed here:

http://www.biomedcentral.com/1471-2466/10/18/prepub

## References

[B1] Frémont B Pacouret G Jacobi D Puglisi R Charbonnier B de Labriolle A Prognostic value of echocardiographic right/left ventricular end-diastolic diameter ratio in patients with acute pulmonary embolism. Results from a monocenter registry of 1,416 patients Chest 2008 133 358 362 10.1378/chest.07-1231 17951624

[B2] Grifoni S Olivotto I Cecchini P Pieralli F Camaii A Santoro G Conti A Agnelli G Berni G Short-term clinical outcome of patients with acute pulmonary embolism, normal blood pressure, and echocardiographic right ventricular dysfunction Circulation 2000 101 2817 2822 10859287 10.1161/01.cir.101.24.2817

[B3] Kucher N Rossi E De Rosa M Goldhaber SZ Prognostic role of echocardiography among patients with acute pulmonary embolism and a systolic arterial pressure of 90 mmHg or higher Arch Intern Med 2005 165 1777 1781 10.1001/archinte.165.15.1777 16087827

[B4] Büller HR Agnelli G Hull RD Hyers TM Prins MH Raskob GE Antithrombotic therapy for venous thromboembolic disease: the Seventh ACCP Conference on Antithrombotic and Thrombolytic Therapy Chest 2004 126 401S 428S 10.1378/chest.126.3_suppl.401S 15383479

[B5] Konstantinides S Geibel A Geusel G Heinrich F Kasper W Heparin plus alteplase compared with heparin alone in patients with submassive pulmonary embolism N Engl J Med 2002 347 1143 1150 10.1056/NEJMoa021274 12374874

[B6] Hamel E Pacouret G Vincentelli D Forissier JF Peijcher P Pottier JM Charbonnier B Thrombolysis or heparin therapy in massive pulmonary embolism with right ventricular dilation. Results from a 128-patient monocenter registry Chest 2001 120 120 125 10.1378/chest.120.1.120 11451826

[B7] Tardy B Venet C Zeni F Coudrot M Guyomar'h S Mismetti P Short term effect of recombinant tissue plasminogen activator in patients with hemodynamically stable acute pulmonary embolism: Results of a meta-analysis involving 464 patient Thromb Res 2009 in press 10.1016/j.thromres.2009.05.009 19493561

[B8] Lankeit M Konstantinides S Thrombolysis for hemodynamically stable patient with pulmonary embolism: Still searching for the intermediate-risk group Tromb Res 2009 in press 10.1016/j.thromres.2009.07.002 19664803

[B9] Smulders YM Pathophysiology and treatment of haemodynamic instability in acute pulmonary embolism: the pivotal role of pulmonary vasoconstriction Cardiovasc Res 2000 48 23 33 10.1016/S0008-6363(00)00168-1 11033105

[B10] Miller RI Das S Anandarangam T Leibowitz DW Alderson PO Thomashow B Homma S Association between right ventricular function and perfusion abnormalities in hemodynamically stable patients with acute pulmonary embolism Chest 1998 113 665 670 10.1378/chest.113.3.665 9515840

[B11] Alpert JS Godtfredsen J Ockene IS Anas J Dalen JE Pulmonary hypertension secondary to minor pulmonary embolism Chest 1978 73 795 797 10.1378/chest.73.6.795 657852

[B12] Halmagyi DF Starzecki B Horner GJ Humoral transmission of cardiorespiratory changes in experimantal lung embolism Circ Res 1964 14 546 552 14169974 10.1161/01.res.14.6.546

[B13] Utsonomiya T Krausz MM Levine L Sherpo D Hechtman HB Thromboxane mediation of cardiopulmonary effects of embolism J Clin Invest 1982 70 361 368 10.1172/JCI110625 6284801 PMC371244

[B14] Utsonomiya T Treatment of pulmonary embolism with prostacyclin Surgery 1980 88 25 30 6992319

[B15] Perlman MB Effects of prostacyclin on pulmonary vascular response to thrombin in awake sheep J Appl Physiol 1986 60 546 553 3512510 10.1152/jappl.1986.60.2.546

[B16] Ueno Y Kawashima A Koike H Nishio S Effect of beraprost sodium, a stable prostacyclin analogue, on pulmonary thromboembolism in mice Thromb Res 1995 77 193 198 10.1016/0049-3848(95)91625-U 7740511

[B17] Webb SAR Stott S Heerden PV The use of inhaled aerosolized prostacyclin in the treatment of pulmonary hypertension secondary to pulmonary embolism Int Care Med 1996 22 353 355 10.1007/BF01700458 8708174

[B18] Mookadam F Jiamsripong P Goel R Warsame TA Emani UR Khandheria BK Critical appraisal on the utility of echocardiography in the management of acute pulmonary embolism Cardiol Rev 2010 18 29 37 10.1097/CRD.0b013e3181c09443 20010336

[B19] Paramothayan NS Lasserson TJ Wells AU Goldhaber SZ Prostacyclin for pulmonary hypertension in adults Cochrane Database Syst Rev (database online) 2005 2 10.1002/14651858.CD002994.pub2 PMC7004255 15846646

[B20] Currie PJ Seward BJ Chan K Fyfe DA Hagler DJ Mair DD Reeder GS Nishimura RA Tajik AJ Continuous wave doppler determination of right ventricular pressure: a simultaneous Doppler-catherization study in 127 patients J Am Coll Cardiol 1985 6 750 756 4031289 10.1016/s0735-1097(85)80477-0

[B21] Konstantinides S Geibel A Olschewski M Heinrich F Grosser K Rauber K Iversen S Redecker M Kienast J Just H Kasper W Association between thrombolytic treatment and the prognosis of hemodynamically stable patients with major pulmonary embolism Circulation 1997 96 882 888 9264496 10.1161/01.cir.96.3.882

[B22] Goldhaber SZ Come PC Lee RT Braunwald D Parker JA Haire WD Feldstein ML Miller M Toltzis R Smith JL Taveira da Silva AM Mogtader A McDonough TJ Alteplase versus heparin in acute pulmonary embolism: randomized trial assessing right ventricular function and pulmonary perfusion Lancet 1993 341 507 511 10.1016/0140-6736(93)90274-K 8094768

[B23] Metz D Nazeyrollas P Mailier B Jennesseaux C Tasson S Maes D Elaerts J Regression of right ventricular hypokinesis after thrombolysis in acute pulmonary embolism Am J Cardiol 1996 77 1252 1254 10.1016/S0002-9149(96)00177-4 8651110

[B24] Come PC Kim D Parker JA Goldhaber SZ Breanwald E Markis JE Early reversal of right ventricular dysfunction in patients with acute pulmonary embolism after treatment with intraveneous tissue plasminogen activator J Am Coll Cardiol 1987 10 971 978 2959713 10.1016/s0735-1097(87)80333-9

[B25] Dias-Junior CA Vieira TF Moreno H Evora PR Tanus-Santos JE Sildenafil selectively inhibits acute pulmonary embolism-induced pulmonary hypertension Pulm Pharmacol Ther 2005 18 181 186 10.1016/j.pupt.2004.11.010 15707852

[B26] Tanus-Santos JE Gordo WM Udelsmann A Cittadino MH Moreno H Nonselective endothelin-receptor antagonism attenuates hemodynamic changes after massive pulmonary air embolism in dogs Chest 2000 118 175 179 10.1378/chest.118.1.175 10893375

[B27] Bottiger BW Motsch J Dorsam J Mieck U Gries A Weimann J Martin E Inhaled nitric oxide selectively decreases pulmonary artery pressure and pulmonary vascular resistance following acute massive pulmonary microembolism in piglets Chest 1996 110 1041 1047 10.1378/chest.110.4.1041 8874266

[B28] Huet Y Brun-Buisson C Lemaire F Teisseire B Lhoste F Rapin M Cardiopulmonary effects of ketanserin infusion in human pulmonary embolism Am Rev Resp Dis 1987 135 114 117 3800140 10.1164/arrd.1987.135.1.114

[B29] Lopez-Candales A Rajagopalan N Saxena N Gulyasy B Edelman K Bazaz R Right ventricular systolic function is not the sole determinant of tricuspid annular motion Am J Cardiol 2006 98 973 977 10.1016/j.amjcard.2006.04.041 16996886

[B30] Capellier G Jacques T Balvay P Blasco G Belle E Barale F Inhaled nitric oxide in patients with pulmonary embolism Int Care Med 1997 23 1089 1092 10.1007/s001340050461 9407246

[B31] Webb SAR Stott S Heerden PV The use of inhaled aerosolized prostacyclin in the treatment of pulmonary hypertension secondary to pulmonary embolism Int Care Med 1996 22 353 355 10.1007/BF01700458 8708174

[B32] Bratel T Lagerstrand L Brodin L Nowak J Randmaa I Ventilation-perfusion relationships in pulmonary arterial hypertension: Effect of intravenous and inhaled prostacyclin treatment Respir Physiol Neurobiol 2007 158 59 69 10.1016/j.resp.2007.03.003 17452130

[B33] Rex S Missant C Segers P Rossaint R Wouters PF Epoprostenol treatment of acute pulmonary hypertension is associated with a paradoxical decrease in right ventricular contractilty Int Care Med 2008 34 179 189 10.1007/s00134-007-0831-8 17710383

[B34] Kerbaul F Brimioulle S Rondelet B Dewachter C Hubloue I Naeije R How prostacyclin improves cardiac output in right heart failure in conjunction with pulmonary hypertension Am J Respir Crit Care Med 2007 175 846 850 10.1164/rccm.200611-1615OC 17272784

[B35] Roeleveld RJ Vonk-Noordegraaf A Marcus JT Bronzwaer JG Marques KM Postmus PE Boonstra A Effects of epoprostenol on right ventricular hypertrophy and dilatation in pulmonary hypertension Chest 2004 12592 572 9 10.1378/chest.125.2.572 14769740

[B36] Montalescot G Drobinski G Meurin P Maclouf J Sotirov I Philippe F Choussat R Morin E Thomas D Effects of prostacyclin on the pulmonary vascular tone and cardiac contractility of patients with pulmonary hypertension secondary to end-stage heart failure Am J Cardiol 1998 82 749 55 10.1016/S0002-9149(98)00439-1 9761085

[B37] O'Grady J Warrington S Moti MJ Effects of intravenous infusion of prostacyclin (PGI2) in man Prostaglandin 1980 19 319 332 10.1016/0090-6980(80)90030-1 6992228

